# Comparison of Different Cell Substrates on the Measurement of Human Influenza Virus Neutralizing Antibodies

**DOI:** 10.1371/journal.pone.0052327

**Published:** 2012-12-20

**Authors:** Weiguo Zhai, Dan Ning Zhang, Cecilia Mai, Justin Choy, Gary Jian, Kuldip Sra, Mark S Galinski

**Affiliations:** 1 Analytical Biochemistry, MedImmune, Mountain View, California, United States of America; 2 Vaccine Analytical Sciences, MedImmune, Mountain View, California, United States of America; Lovelace Respiratory Research Institute, United States of America

## Abstract

Eight cell lines were systematically compared for their permissivity to primary infection, replication, and spread of seven human influenza viruses. Cell lines were of human origin (Caco-2, A549, HEp-2, and NCI-H292), monkey (Vero, LLC-MK2), mink (Mv1 Lu), and canine (MDCK). The influenza viruses included seasonal types and subtypes and a pandemic virus. The MDCK, Caco-2, and Mv1 Lu cells were subsequently compared for their capacity to report neutralization titers at day one, three and six post-infection. A gradient of sensitivity to primary infection across the eight cell lines was observed. Relative to MDCK cells, Mv1 Lu reported higher titers and the remaining six cell lines reported lower titers. The replication and spread of the seven influenza viruses in the eight cell substrates was determined using hemagglutinin expression, cytopathic effect, and neuraminidase activity. Virus growth was generally concordant with primary infection, with a gradient in virus replication and spread. However, Mv1 Lu cells poorly supported virus growth, despite a higher sensitivity to primary infection. Comparison of MDCK, Caco-2, and Mv1 Lu in neutralization assays using defined animal antiserum confirmed MDCK cells as the preferred cell substrate for influenza virus testing. The results observed for neutralization at one day post-infection showed MDCK cells were similar (<1 log_2_ lower) or superior (>1 log_2_ higher) for all seven viruses. Relative to Caco-2 and Mv1 Lu cells, MDCK generally reported the highest titers at three and six days post-infection for the type A viruses and lower titers for the type B viruses and the pandemic H9N2 virus. The reduction in B virus titer was attributed to the complete growth of type B viruses in MDCK cells before day three post-infection, resulting in the systematic underestimation of neutralization titers. This phenomenon was also observed with Caco-2 cells.

## Introduction

Questions have been raised regarding the influenza neutralization assays used by reference laboratories, research centers, and commercial entities [1], [2]. The basis for these concerns is that the incubation time from primary virus infection to result (up to 18 h) is inadequate and should be extended to seven days [1], [2], [3]. The hypothesis is that the short incubation time may not account for the full breadth of immune response and may be primarily associated with an immune response to hemagglutinin (HA). In addition, the preferred cell substrate, Madin Darby canine kidney (MDCK) cells, which are used to report virus infectivity, may have different permissive properties than other cells such as rhesus monkey kidney cells [4], [5]. In response to these questions we investigated the role of cell permissivity and incubation time in neutralization titer.

The influenza Microneutralization (MN) assay has been a standard clinical method for the demonstration of functional serum antibodies following virus infection in humans and animals. Virus-specific neutralization is highly sensitive, strain-specific, and can be completed within a few days. Depending on the assay format, results can be available approximately 24 h post-infection, or alternatively, up to six or seven days post-infection of the reporter cell substrate [6], [7], [8]. Under short incubation times (≤ 24 h), an overlay (agarose or methyl cellulose) is not required, and prevention of primary infection is the principal measure of neutralization. In this assay format the antigen target is the HA protein, and antibodies to HA prevent virus binding, internalization, or uncoating steps of infection. With prolonged incubation (≥ 24 h) without an overlay, prevention of both primary and secondary virus infection, replication and spread are the principal measures of neutralization. In this assay format secondary spread of virus may be blocked by antibodies to the neuraminidase (NA) protein, which may prevent virus progeny release from infected cells. In assays using a prolonged incubation with an overlay (that is, plaque without neutralizing serum in the overlay), neutralization of primary infection is again the principal measure, with the prolonged incubation allowing growth of viral plaques for enumeration.

Influenza MN assay reports the serum dilution that effects a 50% reduction in the measurand relative to a virus control incubated in the absence of antibody (for example, reciprocal log_2_ transformed for 2-fold dilutions). The measurands indicative of virus infection, replication and spread include: staining for influenza proteins (typically, HA, NA and/or nucleocapsid protein) using monoclonal antibodies or polyclonal antiserum; measurement of NA activity; or detection of cytopathic effects (CPE).

A key principle of neutralization assays is that the percentage of measurand reduction is independent of the quantity of virus used in the assay, but dependent on the unit of time used for neutralization. This phenomenon is termed the “percentage law” [9]. The percentage law states that, within certain limits, the same concentration of antiserum will neutralize the same percentage of virus regardless of the amount of virus used in the assay. For assays with an overlay, the time component for neutralization is relatively short (hours) and reflects the time of incubation of virus and antiserum until the removal of sample inoculum and addition of overlay. For assays without an overlay, the time component for neutralization is continuous and spans the entire time of incubation from the mixing of virus and antiserum until assay completion (one to six days). Regardless of assay format, the percentage law is applicable. However, as observed in this study, the percentage law is not valid when neutralization is continuous and virus replication and spread is completed before quantitation of the measurand.

MDCK cells are the preferred cell substrate for analysis of human influenza isolates, as they are highly permissive for human influenza type A and B viruses. MDCK cells are universally used by influenza reference laboratories and research centers [2]. Although other cell lines have been used for influenza virus growth, comparison of the performance characteristics of different cell substrates has not been extensively reported for neutralization assays. In this study eight different cell lines were assessed for permissivity to support influenza virus growth. The cell lines were selected based on their reported capability to support virus infectivity measurements (TCID_50_, plaque assays), diagnostic cell substrates (clinical virus isolation, neutralization assays), virus research, and manufacture of influenza vaccines. Four of the eight cell lines were human, Caco-2 [5], [7], [8], [10], A549 [5], [11], [12], HEp-2 [13], and NCI-H292 [11], [14], [15]; two were monkey, LLC-MK2 [6], [16], [17], and Vero [18], [19], [20]; one was mink, Mv1 Lu [21], [22], [23]; and one was canine, MDCK [3], [5], [11], [24].

The eight candidate cell lines were assessed systematically for their sensitivity to primary influenza virus infection, and their permissivity for virus replication and spread. Seven different influenza viruses, including three subtypes of influenza type A (H1N1, H3N2, H9N2) and two lineages (Victoria and Yamagata) of influenza type B were studied. Sensitivity was defined as the ability of a cell line to report infectious particles relative to MDCK cells at one day post-infection. Permissivity was defined as the ability of limiting particle numbers to replicate and spread through the cell substrate similar to MDCK cells over six days. Together, sensitivity and permissivity were considered important virological features for defining a cell substrate as fit for use in a neutralization assay.

Three of the eight cell lines were compared as cell substrates in MN assays. MN analysis included neutralization with defined control animal sera (anti-HA, anti-NA from sheep and anti-influenza virus from ferret). The neutralization was assessed at one, three, and six days post-infection to determine the effect of incubation time on neutralization titers. The MDCK cells were determined to be fully permissive for all influenza viruses and were the most consistent cell substrate for reporting MN titers for all influenza viruses tested.

## Results

### Cell Line Screening: Sensitivity to Primary Infection

**Figure 1:**
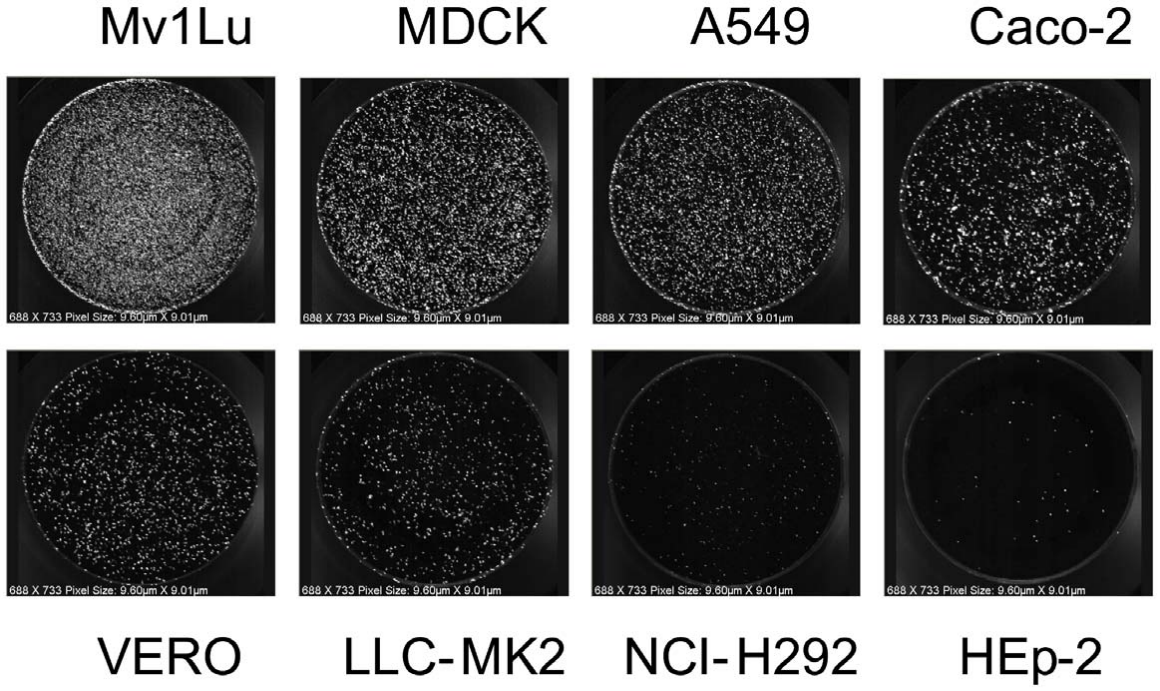
Sensitivity of eight cell lines to primary infection by A/Uruguay/716/07. Cell lines Mv1 Lu, MDCK, Caco-2, A549, Vero LLC-MK2, NCI-H292, and HEp-2, were infected with the same serial 10-fold dilution of A/Uruguay/716/07 at ∼10,000 FFU per well (FFU based on infectivity in MDCK cells). Plates were incubated at 33°C for 18 to 22 h post-infection, then stained using strain-specific sheep polyclonal anti-HA antibody.

The sensitivity of eight cell lines to various influenza virus infection (two H1N1, two H3N2, one H9N2, and two B strains) was determined using a potency assay, the fluorescent focus assay (FFA), on Day 1 post-infection. **Table 1** summarizes the absolute and relative potency titers observed in each cell line for each virus. Because the input viral HA was already proteolytically cleaved, and the potency was determined at 18 to 22 h post-infection, no differences in potency due to addition of trypsin were observed (data not shown), confirming that the infectivity titer is a measure of primary infection. The FFA potency data normalized relative to MDCK cells showed that Mv1 Lu cells were more sensitive to all of the virus types/subtypes, by up to 0.5 log_10_ (3-fold) higher in infectivity, and that HEp-2, LLC-MK2, and NCI-H292 cells would in general show a 1.0 log_10_ or greater (10-fold) reduction compared with MDCK cells. **Figure 1** shows an example of foci density for A/Uruguay/716/07 (H3N2) in the 8 cell lines at ∼10,000 focus forming units (FFU)/well. The foci density spans an obvious gradient across the different cell lines, demonstrating a rank order of sensitivities to primary infection. Similar observations were made for the other influenza virus types/subtypes (data not shown). Based on these observations, Mv1 Lu and MDCK were determined to be the most sensitive cell lines to primary influenza virus infection, with Mv1 Lu showing reproducibly higher titers.

**Table 1:** Analysis of primary infection of seven different influenza virus types/subtypes in eight different cell lines.

Potency^a^	Virus	MDCK	Mv1 Lu	Caco-2	A549	Vero	LLC-MK2	NCI-H292	HEp-2
Absolute	A/CA	9.0	9.5	8.4	8.5	7.9	7.3	6.8	NR^b^
	A/HK	8.7	8.8	7.8	7.8	7.7	7.3	6.9	NR
	A/PTH	8.9	9.1	8.5	8.9	8.2	7.9	7.8	6.8
	A/SD	9.0	9.2	8.4	8.6	7.9	7.5	7.2	NR
	A/UR	9.0	9.5	8.1	8.7	8.3	7.9	7.9	6.8
	B/FL	9.1	9.5	8.7	8.9	9.0	8.5	7.7	NR
	B/MAL	9.3	9.8	8.7	9.1	9.0	8.4	7.6	NR
Relative^c^	A/CA	–	0.5	−0.6	−0.5	−1.1	−1.7	−2.2	NR
	A/HK	–	0.1	−0.9	−0.9	−1.0	−1.4	−1.8	NR
	A/PTH	–	0.3	−0.4	0.0	−0.7	−1.0	−1.0	−2.1
	A/SD	–	0.2	−0.6	−0.4	−1.1	−1.5	−1.8	NR
	A/UR	–	0.5	−0.9	−0.3	−0.7	−1.1	−1.1	−2.3
	B/FL	–	0.4	−0.5	−0.2	−0.1	−0.7	−1.4	NR
	B/MAL	–	0.4	−0.7	−0.2	−0.3	−0.9	−1.7	NR

a Potency determined at Day 1 post-infection as log_10_ FFU per mL, representing the average of three testing days.

b NR = Not Reported, as the foci count was below the limit of quantitation (<15 foci).

c Potency relative to MDCK cells.

### Cell Line Screening: Sensitivity to Replication and Spread

**Figure 2:**
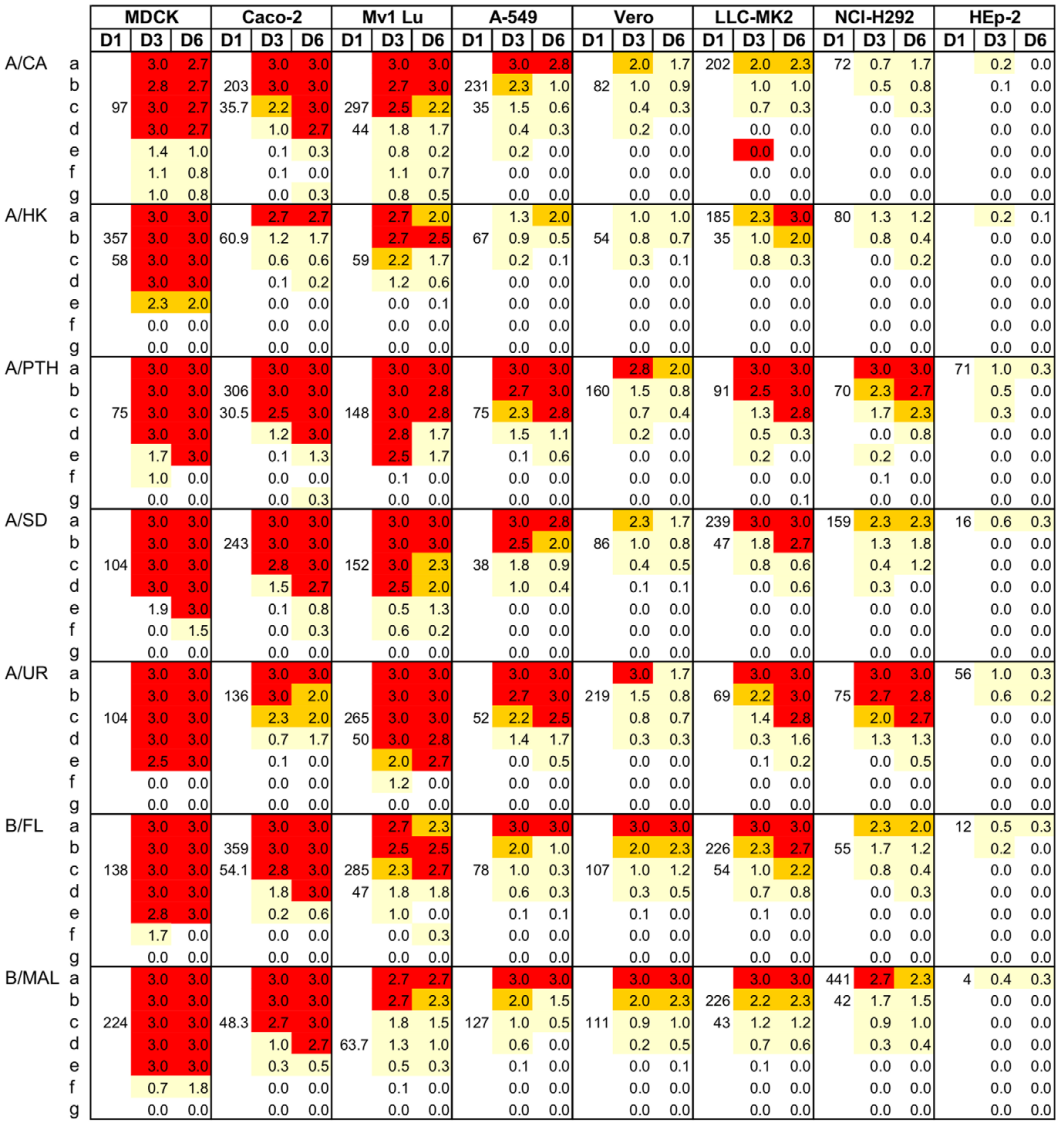
Composite analysis of replication and spread of seven different influenza types/subtypes in eight different cells. The A549, Caco-2, HEp-2, LLC-MK2, MDCK, Mv1 Lu, NCI-H292, and Vero cells were infected with the same serial 10-fold dilutions (for virus nomenclature see Materials and Methods) to limiting dilution starting at approximately 10,000 FFU per well (FFU based on infectivity in MDCK cells). Composite data are for FFA foci number on Day 1 (D1) and HA expression at Day 3 (D3) and 6 (D6) post-infection. The results represent the average of 3 independent assays. Culture medium was supplemented with TPCK-Trypsin at 0.5 µg/ml. The FFA foci number is the average number of foci over 3 independent assays and indicates the row used for counting foci. Each row (a-g) represents a 10-fold dilution of virus and rows below the foci count would contain progressively fewer infectious particles until the dilution limit is reached. Rows above the countable foci had too many foci to accurately count and row H was used as blank. Average subjective scores (see Materials and Methods) were determined (values of 0, 0.5, 1, 2, and 3) and color coded as white (0 no HA expression), cream (0.1–1.9), orange (2–2.4) and red (2.5–3.0).

The ability of the seven influenza types/subtypes to replicate and spread in the eight cell substrates was determined by assessing HA antigen expression, CPE, and NA activity on Days 3 and 6. The results for HA expression in the different cell lines are shown in **Fig. 2**. This figure is a composite heat map for visualization of the data matrix and includes the eight cell lines and seven influenza viruses at Days 1, 3, and 6 (D1, D3, and D6, respectively). Serial 10-fold dilutions (indicated as rows a-g) of each virus starting at ∼10,000 FFU (in MDCK units) per well, were used to infect specific cell lines in 96-well plates. The number shown under each cell line D1 represents the infected cell foci count per well at the dilution used for calculation of the titers in **Table 1**. Rows above this number had too many foci to count (each row is 10-fold higher) and rows below this number had 10-fold fewer foci until limiting dilution was obtained. The average of three independent assay days for the HA antigen content are reported numerically at Day 3 and Day 6 for each dilution, and color coded as white (0 no HA expression), cream (0.1–1.9 or equivalent of a subjective+score), orange (2–2.4 or equivalent of a subjective++score) and red (2.5–3.0 or equivalent of a subjective+++score).

**Figure 3:**
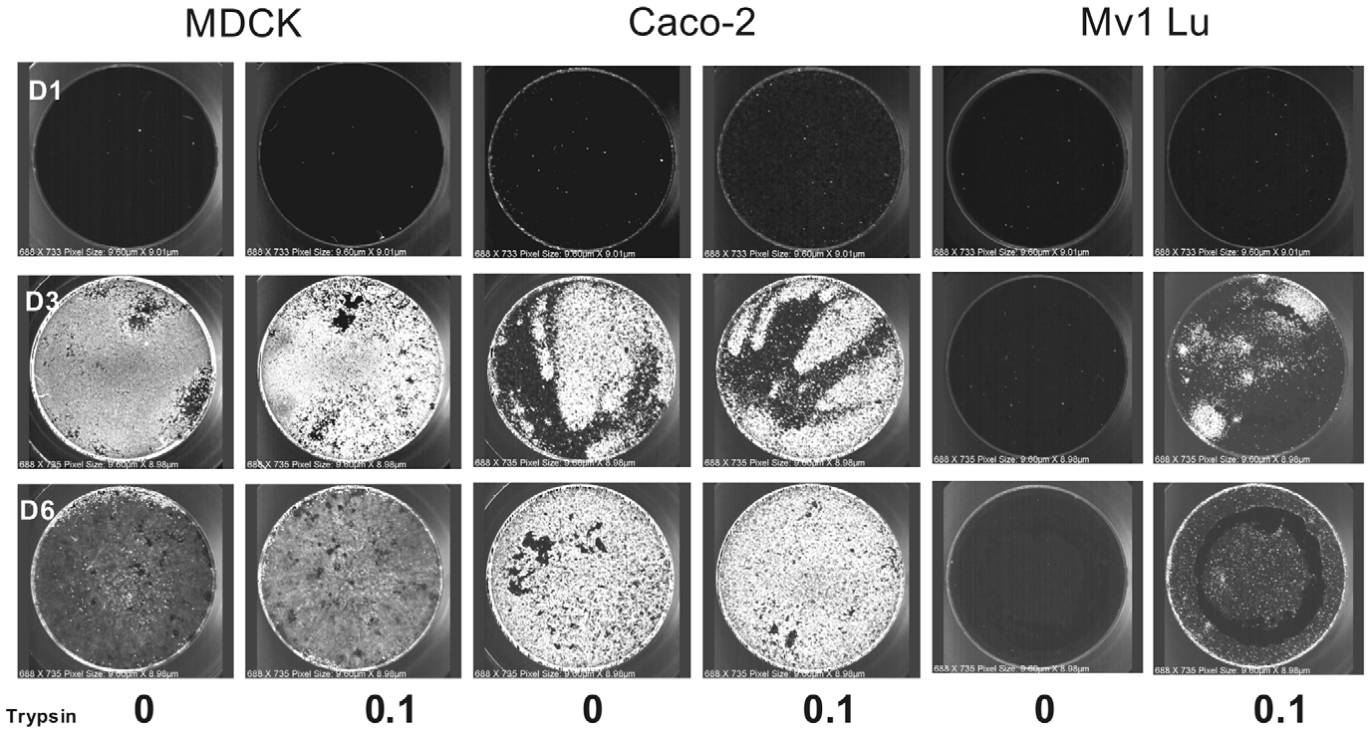
Trypsin-independent replication and spread of B/Florida/04/06 in MDCK, Caco-2, and Mv1 Lu cells. Cell lines MDCK, Caco-2, and Mv1 Lu were infected with the same serial 10-fold dilution of B/Florida/04/06 starting at approximately 10,000 FFU per well (FFU based on infectivity in MDCK cells). Cells were supplemented with 0.1 µg/ml or no trypsin (as indicated) and were incubated at 33°C for 18 to 22 h post-infection and stained using strain-specific sheep polyclonal anti-HA antibody. The wells shown above are from a dilution that contained approximately 10 (MDCK cells) or 40 (Caco-2 and Mv1 Lu cells) infectious particles on Day 1, 3, and 6 post-infection.

**Figure 2** extends the observations of the Day 1 FFA determinations (**Table 1**), and demonstrates that there was a gradient in the cell lines to support virus replication and spread. In particular, at limiting dilution where a single or a few infectious particles were introduced, virus replication and spread is readily observed by HA antigen expression. For type A viruses, trypsin supplementation is required (data not shown), whereas for type B viruses, trypsin was not required (**Figure 3**).

The Caco-2 cells showed a reduced sensitivity to infection compared with MDCK cells (**Table 1**), but supported all seasonal influenza viruses (H1N1, H3N2, and B) replication as determined by HA expression and NA activity (**Figure 2** shows HA activity). The Caco-2 cells had a significantly reduced sensitivity to primary infection with the H9N2 A/HK on Day 1 (0.9 log_10_ lower than MDCK cells) and a reduced efficiency to support A/HK replication on Day 3 and Day 6. As noted, the Mv1 Lu cells had the highest sensitivity to primary infection (**Table 1**) for all influenza virus types/subtypes on Day 1 compared to MDCK cells. However, virus replication and spread in Mv1 Lu cells was significantly diminished at Day 3 and Day 6 post-infection for all viruses as determined by reduced HA expression and NA activity (**Figure 2** shows HA activity), in particular for A/HK, B/FL, and B/Mal viruses.

**Figure 3** shows an example of HA antigen expression for B/Florida/04/06 in MDCK, Caco-2, and Mv1 Lu cells at input particles per well of 10, 40, and 40, respectively, at days 1, 3, and 6. This figure demonstrates several key features: (1) robust virus spread from a few particles to encompass the entire monolayer occurs rapidly for MDCK and Caco-2 cells, whereas replication and spread is significantly diminished in Mv1 Lu cells; (2) virus replication and spread does not require trypsin for type B viruses; and (3) in MDCK cells, virus infection is completed before Day 3 with subsequent loss of the monolayer, while in Caco-2 cells the monolayer remains intact despite complete virus replication by Day 6.

With the exception of A549 cells, the remaining cell lines (Vero, LLC-MK2, NCI-H292, and HEp-2) had a much lower sensitivity to primary infection (**Table 1**) and did not meet pre-specified acceptance criterion (Day 1 titer no less than 1.0 log_10_ relative to MDCK cells for all virus types and subtypes) for progression to the next assessment stage. In addition, all of these other cell lines, including A549, did not support replication and spread of influenza types/subtypes as determined by HA expression and NA activity at Day 3 and Day 6 post-infection and (**Figure 2** for HA expression), and thus were not evaluated in the neutralization assay.

### Cell Line Screening: Cytopathic Effect (CPE)

The assessment of CPE was, in general, consistent with HA expression. However, difference in CPE degree was observed across cell lines and HA expression was determined to be a more reliable attribute to measure than CPE. In particular, Caco-2 cells did not demonstrate significant CPE unless the well was infected at high particle numbers and degradation of the monolayer was complete (data not shown). In contrast, at limiting dilution HA expression was observed throughout the Caco-2 cell monolayer (**Figure 3**), even if CPE was negligible. Therefore, CPE was considered a poor attribute for measuring virus replication and spread in these cells.

### Cell Line Screening: Neuraminidase (NA) Activity

**Figure 4:**
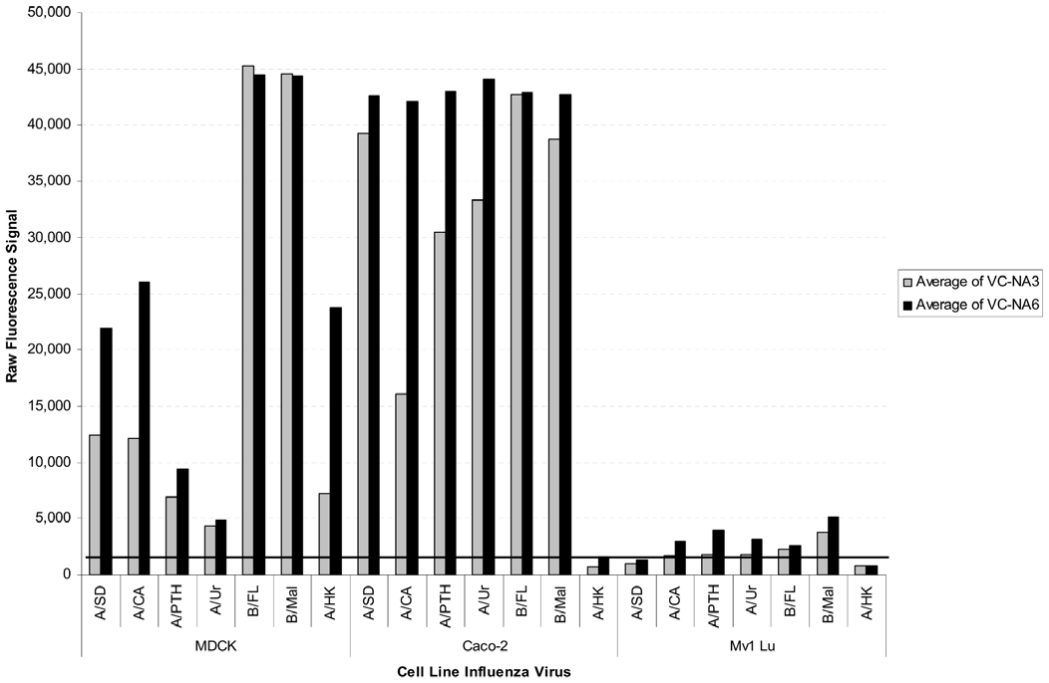
NA activity in virus infected control spent supernatants at day 3 and 6 post-infection. Approximately 100 FFU of each virus type/subtype (see Materials and Methods for virus nomenclature) were used in neutralization assays. Spent culture medium supernatants from un-neutralized virus control wells were harvested on Day 3 (gray) and Day 6 (black) and tested for NA activity. The raw fluorescence signals are plotted for each type/subtype by cell line (MDCK, Caco-2, and Mv1 Lu). The NA activity presented is the average of 3 test days. The solid black line indicates the cell control threshold for determining whether virus replication was reportable above background.

NA activity was also measured to demonstrate that virus release into the culture medium could be measured. Insufficient virus replication occurs by Day 1 and NA activity was not reliably detected above the cell control signal, even at ∼10,000 FFU per well (data not shown). However, as shown in **Figure 4**, NA activity was clearly measurable on Day 3 and 6, with good concordance to the observed HA expression in the wells for MDCK, Caco-2, and Mv1 Lu cells. Similar results were also obtained for LLC-MK2, Vero, HEp-2, and NCI-H292 cells, but at a comparably low level as was observed for HA expression (data not shown). The recovery of NA activity in the supernatant suggested that influenza virus particles were secreted into the culture medium at sufficient quantities and that either HA expression or NA activity could be used to demonstrate the replication and spread of influenza virus in cells at limiting dilution. Interestingly, A/HK replication and spread was greatly diminished in Caco-2 and Mv1 Lu cells relative to MDCK.

### Microneutralization (MN): Comparison of MDCK, Caco-2 and Mv 1 Lu Cells

The MDCK, Caco-2 and Mv1 Lu cells were further assessed in the MN assays. Caco-2 and Mv1 Lu cells had similar sensitivity to influenza infection and reported titers that were ±1 log_10_ of those observed in MDCK cells (**Table 1**). In addition, they were sufficiently permissive to support replication and spread of most of the influenza types/subtypes at limiting particle numbers (**Figure 2**). The MN assays used defined control sera that block virus attachment (using anti-HA hyperimmune sheep serum from MedImmune, aHA), virus spread (using anti-NA hyperimmune sheep antiserum from NIBSC, aNA) and more broadly to all immunologically relevant proteins with polyclonal antibody (using in-house virus vaccinated ferret serum, fPost).

**Figure 5:**
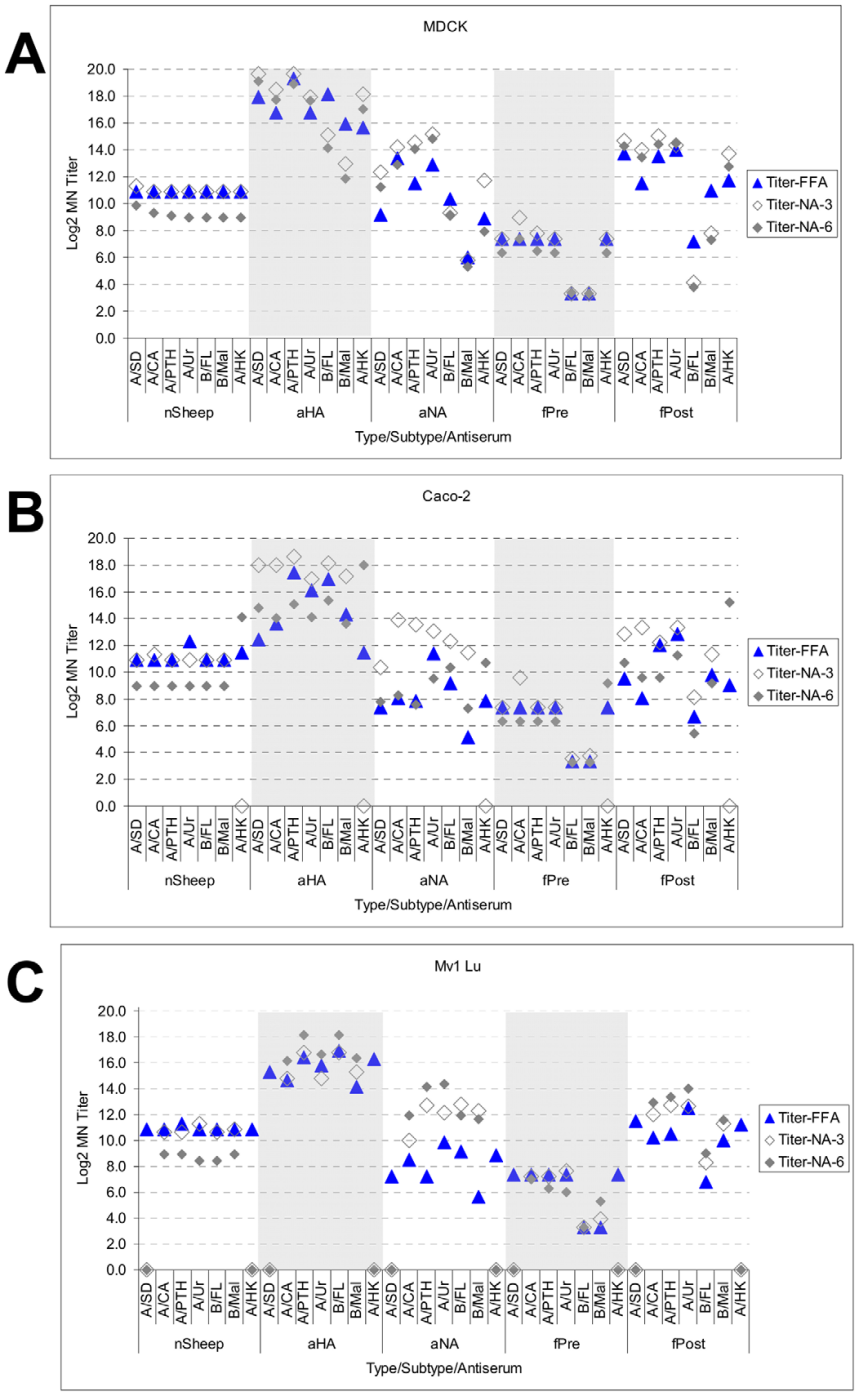
Neutralization of seven different influenza virus types and subtypes in MDCK, Caco-2, and Mv1 Lu cells (by cell line). Approximately 100 FFU of each virus type/subtype (see Materials and Methods for virus nomenclature) was added to serial 2-fold dilution of normal sheep antiserum (nSheep), sheep anti-HA (aHA), sheep anti-NA (aNA), or ferret polyclonal antiserum (pre- [fPre] or post-immunization [fPost] with the specified virus strain) and incubated at room temperature for 30 min. The serum treated virus was transferred onto MDCK (panel A), Caco-2 (panel B) and Mv1 Lu (panel C) cells. Neutralization titers were determined by FFA on Day 1 (blue solid diamonds, Titer-FFA) and NA activity on Day 3 (grey open diamonds; Titer-NA-3) and Day 6 (solid grey diamonds; Titer-NA-6). Titers were determined based on the log_2_ dilution that reduced the foci number or NA activity greater than or equal to 50% of that observed in virus control wells. Titers at the baseline (0.0) represent non-reportable titers, as virus growth was insufficient to make an estimate. The Day 1 and Day 3 titers are the average of 3 assays and the Day 6 titers are the average of 6 assays. The nSheep serum had a titer of <6.3 when assayed with minimal dilution. The titer reported in this chart is higher due to the starting dilution used in assays that was identical to the dilution used for the hyperimmune anti-HA antisera.

**Figure 6:**
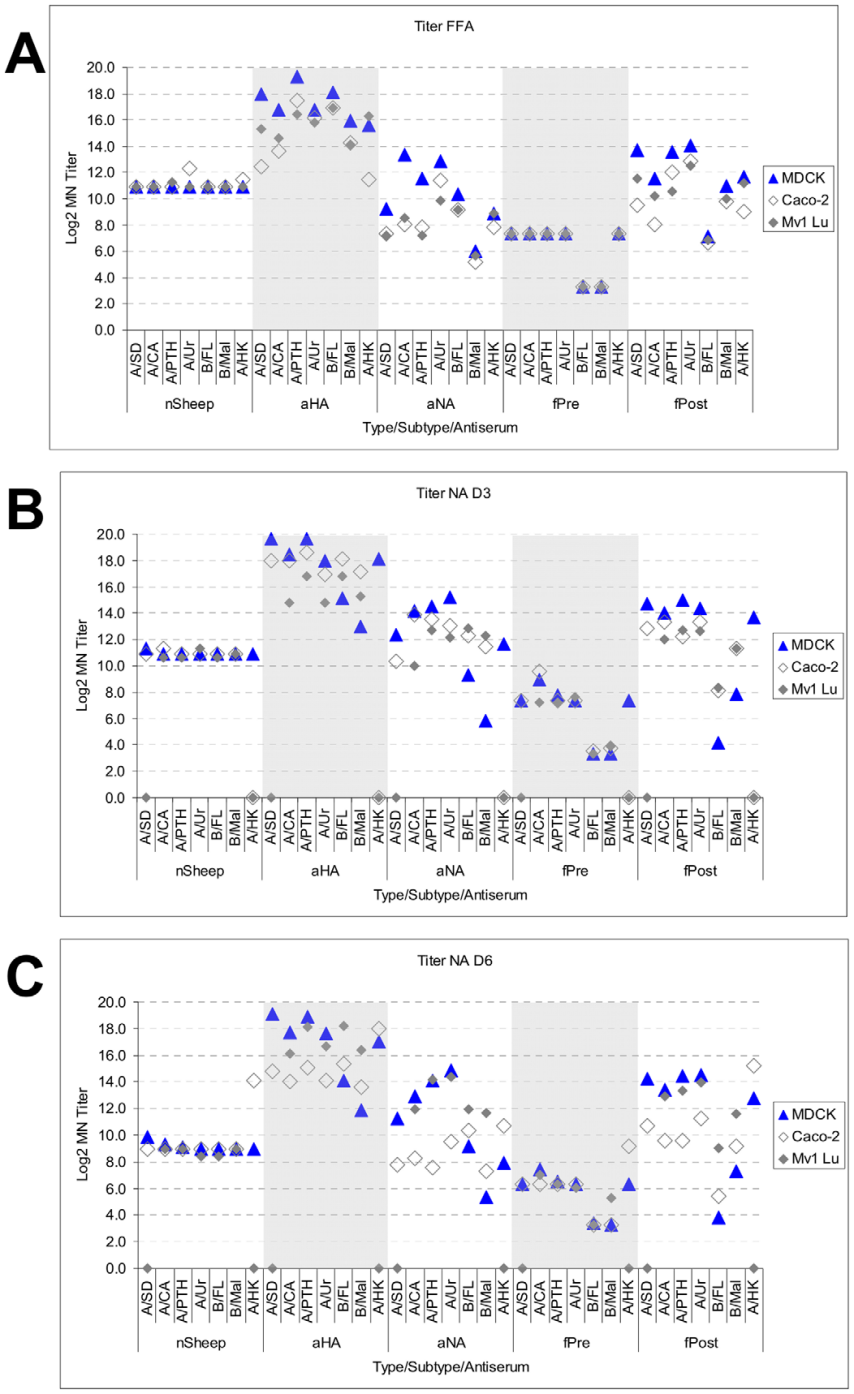
Neutralization of seven different influenza virus types and subtypes in MDCK, Caco-2, and Mv1 Lu cells (by days). Neutralization titers in MDCK (blue solid triangles), Caco-2 (gray open diamonds), and Mv1 Lu (gray solid diamonds) cells were determined by FFA at Day 1 (panel A), and NA activity at Day 3 (panel B) and Day 6 (panel C). Approximately 100 FFU of each virus type/subtype were added to serial 2-fold dilution of normal sheep antiserum (nSheep), sheep anti-HA (aHA), sheep anti-NA (aNA), or ferret polyclonal antiserum (pre- [fPre] or post-immunization [fPost] with the specified virus strain) and incubated at room temperature for 30 min. Serum treated virus was then transferred onto cells and titers were determined by NA activity. Titers were determined based on the log_2_ dilution that reduced the NA activity greater than or equal to 50% of that observed in virus control wells. The nSheep serum had a titer of <6.3 when assayed with minimal dilution. The titer reported in this chart is higher due to the starting dilution used in assays that was identical to the dilution used for the hyperimmune anti-HA antisera.

The results for the MN titers observed in each cell line on Day 1, Day 3, and Day 6 post-infection are shown in **Figure 5** and **Figure 6**. As shown in **Figure 5A** for MDCK cells, the MN titers (using defined control sera) for type A viruses (H1N1, H3N2, and H9N2) on Day 3 and Day 6 (as measured by NA activity) were higher than those observed on Day 1 (as measured by FFA) by approximatley 2- to 4-fold. In contrast to the type A virus titers, the MN titers observed for the type B viruses in MDCK cells were lower on Day 3 than on Day 1 and continued to decline on Day 6.

The naive Sheep (nSheep) and pre-immune ferret (fPre) antisera did not show marked difference in MN titers between Day 1 and Day 6 as expected. It should be noted that the nSheep antisera had a titer (log_2_) of <6.3 when assayed at a starting dilution of 1∶80. The MN titers shown in **Figure 5** and **Figure 6** represent the average starting dilution for all assay days, including starting dilutions that were equivalent in dilution to the hyper-immune anti-HA antisera (1∶5120) and not due to pre-existing antibody.

**Figure 5B** shows the neutralization titers on Day 1, Day 3, and Day 6 in Caco-2 cells. In contrast to the MN titers observed in MDCK cells, both type A (except for H9N2) and type B viruses had higher titers on Day 3 than Day 1. However by Day 6 the MN titers were comparable or lower than the Day 1 titers. Similar to observations in MDCK cells, maximum virus growth in Caco-2 cells (as measured by NA-activity) occurred before Day 6 (**Figure 4**) and the extended incubation (Day 6) reduced the titer from Day 3 for all viruses. It should be noted that A/HK did not have reportable MN titer on Day 3 for any of the antisera due to poor growth, but had a measurable MN titer on Day 6 that exceeded the Day 1 titer and contrasted with the decline observed for all of the other viruses.

**Figure 5C** shows the neutralization titers on Day 1, Day 3, and Day 6 in Mv1 Lu cells. While MN titers were obtained on Day 1 for all viruses, A/HK and A/SD did not have reportable titer on Day 3 or Day 6 for any of the antisera due to poor growth based on NA activity and HA expression. For the remaining viruses, there was a titer increase on Day 3 that, in general, continued to rise by Day 6.

The neutralization titers observed in the three cells lines in **Figure 5** are re-presented in **Figure 6** to compare each cell line at each day post-infection. As shown in **Figure 6**, all cell lines had measurable MN titers on Day 1 by FFA, and MDCK cells generally showed the highest MN titer across all virus types/subtypes (up to 16-fold higher). The MN titers observed for MDCK cells on Day 3 were generally higher for all type A viruses, but lower for type B lineages. As discussed above, the decline in MN titers for type B viruses is attributed to the rapid replication and spread of virus, which is essentially complete before Day 3 post-infection. Mv1 Lu cells did not have reportable MN titers for A/SD and A/HK, and Caco-2 cells did not have reportable MN titers for A/HK, both due to poor virus growth. The MN titers observed for MDCK cells on Day 6 were generally higher for type A viruses, but lower for type B lineages. As discussed above the decline in MN titers for type B viruses is attributed to the rapid replication and spread of virus, which is essentially complete before Day 3 post-infection. Caco-2 cells showed a higher MN titer for A/HK on Day 6. This lagging robust titer was likely an artifact of poor A/HK growth resulting in an increased susceptibility to inhibition by the neutralizing antiserum and not due to measurement of another neutralizing property of the antiserum.

The neutralization titers on Day 6 post-infection are summarized in **Table 2** for all of the defined control antisera; the geometric mean fold rise (MFR) from the normal sheep serum (using the minimum starting dilution tested) and ferret pre-immune sera are included. Our data show that MDCK cells reported the highest MN titers and MFR for the type A viruses. The Mv1 Lu cells appeared to give the highest MN titers and MFR on Day 6 post-infection for the type B viruses (B/FL and B/MAL) despite the poor growth of influenza types and subtypes in this cell line. These apparent results must be considered in relationship to the time course of virus replication, which was essentially completed before Day 3 in MDCK cells and between Day 3 and Day 6 in Caco-2 cells, resulting in a measured MN titer reduction on Day 6 for these cells.

**Table 2:** Neutralization Titers (GMT) on Day 6 Post-Infection and Geometric Mean Fold Rise (MFR) for Defined Control Sera.

Cell Line	Virus	nSheep a,b	aHA^a^	MFR aHA	aNA^a^	MFR aNA	fPre^a^	fPost^a^	MFR fPost
**MDCK**	A/SD	5.3	19.1	13.8	11.3	6.0	6.3	14.3	7.9
	A/CA	5.3	17.7	12.4	12.9	7.6	7.4	13.4	6.0
	A/PTH	5.3	18.9	13.6	14.1	8.8	6.5	14.4	7.9
	A/UR	5.3	17.6	12.3	14.8	9.5	6.3	14.5	8.2
	B/FL	5.3	14.1	8.8	9.2	3.9	3.4	3.8	0.4
	B/MAL	5.3	11.9	6.6	5.3	0.0	3.3	7.3	4.0
	A/HK	5.3	17.0	11.7	7.9	2.6	6.3	12.8	6.4
**Caco-2**	A/SD	5.3	14.8	9.5	7.8	2.5	6.3	10.7	4.4
	A/CA	5.3	14.0	8.7	8.3	3.0	6.3	9.6	3.3
	A/PTH	5.3	15.0	9.7	7.6	2.3	6.3	9.6	3.3
	A/UR	5.3	14.1	8.8	9.5	4.2	6.3	11.3	4.9
	B/FL	5.3	15.4	10.1	10.3	5.0	3.3	5.4	2.1
	B/MAL	5.3	13.6	8.3	7.3	2.0	3.3	9.2	5.9
	A/HK	5.3	18.0	12.7	10.7	5.4	9.2	15.2	6.0
**Mv1 Lu**	A/SD	5.3	NR^c^	NR	NR	NR	NR	NR	NR
	A/CA	5.3	16.1	10.8	11.9	6.6	7.0	12.9	5.9
	A/PTH	5.3	18.1	12.8	14.2	8.9	6.3	13.3	7.0
	A/UR	5.3	16.7	11.4	14.4	9.1	6.0	14.0	8.0
	B/FL	5.3	18.2	12.9	11.9	6.6	3.3	9.0	5.7
	B/MAL	5.3	16.4	11.1	11.7	6.4	5.3	11.6	6.3
	A/HK	5.3	NR	NR	NR	NR	NR	NR	NR

a MN Titers were calculated as the average of 6 assay days.

b nSheep serum neutralization titer has reportable values of <6.3 and are assigned 5.3 for calculation of MFR.

c NR = Not reportable as virus control NA activity was less than 2.5-fold of the cell control NA activity.

## Discussion

The primary objective of this study was to increase understanding of the principles of MN and its potential application to serological diagnosis, and not specifically for the analytical optimization of the assay method. More specifically, this study examined the properties of eight different cell lines to support human influenza virus infection, replication and growth, with the intent to identify cell lines with appropriate virological attributes to assess further in MN assays. As MDCK cells are the preferred cell substrate for neutralization assays, the experiments used MDCK cells as the standard for comparison.

The approach that was used included the assessment of cell line sensitivity to primary infection and cell line permissivity to support replication and spread of virus progeny. Sensitivity was defined as the ability of a cell line to report infectious particles relative to MDCK cells at one day post-infection, while permissivity was defined as the ability of limiting particle numbers to replicate and spread through the cell substrate with properties similar to MDCK cells over six days. These attributes were considered important virological features for defining a cell substrate as fit for use in a neutralization assay.

Our results demonstrate that there was a gradient of sensitivity across the eight cell lines, with one cell line (Mv1 Lu) reporting higher titers (0.1 to 0.5 log_10_ ) relative to MDCK cells, and the remaining six cell lines reporting lower titers (0.0 to −2.2 log_10_) relative to MDCK cells (**Table 1**). Similarity to MDCK cells in sensitivity was considered an important attribute since, with decreasing sensitivity, fewer infectious units were observable in the less sensitive cell substrate. Since the comparative neutralization experiments intended to use the same number of infectious units (∼100 FFU based on MDCK cells), differences of greater than 1.0 log_10_ were considered a potential source of systematic bias that would impact the observed results. Although cell line-specific infectious units could have been used (for example, ∼100 MDCK infectious units compared to ∼100 Caco-2 infectious units), the approach considered any cell line as a reporter system for measuring virus replication (that is, neutralization). The input infectivity was, therefore, based on the same number of infectious particles, defined in this study using MDCK cell infectivity.

A gradient in permissivity was also observed across the various cell lines assessed. With decreasing sensitivity to infection, there was also a decrease in the ability of a few infectious particles to replicate and spread through the monolayer relative to MDCK cells. Of particular note was the reduced permissivity of Mv1 Lu cells, which demonstrated the highest sensitivity for infection. Based on HA expression and NA activity, most influenza viruses replicated marginally or not at all over the six days of incubation in A549, Vero, LLC-MK2, NCI-H292, and HEp-2 cells.

The comparative neutralization experiments confirmed the established utility of MDCK cells as the preferred cell substrate for influenza virus testing. The results observed for neutralization at one day post-infection by FFA analysis showed that MDCK cells were similar (<1 log_2_ lower) or superior (>1 log_2_ higher) to Caco-2 and Mv1 Lu cells for all seven viruses using specific animal antiserum that block virus attachment (anti-HA antiserum), virus release from infected cells (anti-NA antiserum) or polyvalent antiserum (ferret anti-influenza antiserum). Under this assay format, primary infection is trypsin independent as the viral HA in the inoculum is already prototypically processed (uncleaved precursor, HA0, to cleaved disulfide linked HA1-HA2), and neutralization is thought to be a measure of inhibition of virus binding, internalization, or uncoating steps of infection. While measurable titers were observed for anti-NA antisera by FFA analysis, this observation is likely attributable to the presence of low levels of cross-reactive HA antibodies as demonstrated by low but measurable HAI titers using the heterologous strains as antigen (data not shown). Thus the H7N1 and H7N2 reassortant used to prepare the anti-N1 and anti-N2 antisera had detectable cross-reactive HAI titers to H1N1, H2N2, and H9N2 strains used in this study. In addition, the influenza B lineage specific anti-NA sheep antisera were prepared from purified NA proteins that may have contained residual co-purifying HA. Despite differences in sensitivity between MDCK, Caco-2, and Mv1 Lu cells, neutralization of primary infection using MDCK cells appears to be the most sensitive cell substrate for determining these titers.

In continuous neutralization assay formats that use longer incubation time post-infection, differences in neutralization titers were observed. In general, MDCK cells reported the highest titers, relative to Caco-2 and Mv1 Lu cells, at three and six days post-infection for the seasonal type A influenza strains (H1N1 and H3N2) and lower titers for the type B viruses and the pandemic H9N2 virus. The decline in MN titer for the type B viruses in MDCK cells may be attributed to the fact that replication and spread of B viruses were already completed by Day 3 in the virus infected control wells (**Figure 4**) and, thus, the maximum level of NA activity was reached before Day 3. In wells containing virus and diluted neutralizing antiserum (sufficient to cause >50% inhibition of virus growth) continued virus growth would result in increased NA activity until it exceeded the 50% threshold required for defining a 50% reduction, resulting in an apparent decline in neutralizing titer relative to the virus infected control wells. This apparent decline continued with incubation and Day 6 titers were even lower than Day 3 titers due to increasing NA activity in antiserum-containing wells.

Interestingly, despite the decreased permissivity of Mv1 Lu cells, neutralization titers were measurable for all viruses that replicated in Mv1 Lu, and for type B viruses, Mv1 Lu cells reported the highest neutralization titers at day six post-infection. The higher titer for type B viruses reflected the poor virus replication in these cells compared to MDCK and Caco-2 cells and not due to measurement of another neutralizing property of the antiserum.

The results observed for the comparative neutralization experiments are best understood within the context of the percentage law and the continuous neutralization format of the assay. The percentage law states that, within certain limits, the same concentration of antiserum will neutralize the same percentage of virus regardless of the amount of virus used in the assay, including continuous neutralization over time where different cell substrates effectively produce different amounts of virus progeny. As noted the MDCK cells and Caco-2 cells are highly permissive for virus replication and spread, despite a modestly lowered sensitivity for Caco-2 cells for all viruses studied. In contrast, Mv1 Lu was poorly permissive despite its high sensitivity. Together these cell lines demonstrated a gradient of virus productivity that reflects different amounts of virus at a fixed concentration of antiserum. In theory, whether the cells are highly or poorly permissive, the neutralization titers should be similar. Indeed, with poorly permissive cells, Mv1 Lu in general, and Caco-2 for H9N2, neutralization titers were measurable and in some instances even higher than the neutralization titers from MDCK cells (that is, Mv1 Lu for all B viruses and Caco-2 for A/HK).

The apparent improved sensitivity, where Mv1 Lu or Caco-2 cells report higher titers than MDCK cells, is attributed to the time when the measurand is quantified (incubation time). As the time point to measure was after complete virus replication, the percentage law is no longer valid as the virus control reached a maximum (for example, NA activity) before the actual measurement. In wells that contain antiserum, neutralization occurred with continuous virus growth. Relative to the virus control, which was fixed at a maximum, the percentage of virus growth (reciprocal of neutralization) relative to the virus control becomes greater and greater, subsequently exceeding 50% neutralization with time. This increase results in assigning a lower titer (that is, dilution) and an apparent fall in the neutralization titer.

In summary, MDCK cells are fully permissive for influenza virus infection, replication and spread. These cells were found to be the only cell line that consistently replicate all viruses (H1N1, H3N2, H9N2, and B) while both Caco-2 and Mv1 Lu cells were found to be deficient in that they do not support growth of all virus types and subtypes. Additionally, Mv1 Lu cells were poorly permissive for virus replication and spread in general. While a 6 day neutralization assay gives improved sensitivity for type A viruses (4-fold higher titer) in MDCK, the prolonged incubation biases B virus titers to be lower than the titer observed on Day 1 due to the rapid growth of these viruses, which is essentially completed on or before Day 3. This phenomenon was also observed with Caco-2 cells; however, the growth of B viruses in these cells was completed between Day 3 and Day 6, resulting in a reduced titer at Day 6 only.

The report from a scientific workshop on serology assays and correlates of protection for influenza vaccines, EMA/732806/2010 [1], suggested that the currently used MN assay is not adequate and a “test with long incubation time (>5 days) needs to replace the short-term culture”. While there may be merit in extending the incubation time for type A viruses, our observations indicate that an incubation of less than 3 days is preferred for B viruses to reach an optimal MN titer. Therefore, a rapid assay using NA activity as a measure of influenza type B virus replication and spread could be implemented with a shortened incubation period of less than 3 days.

This study demonstrated that despite the poor growth of influenza in Mv1 Lu cells, the microneutralization assay is remarkably robust. Thus, even a poorly permissive cell substrate like Mv1 Lu cells can be used to measure neutralizing antibodies if a sufficiently sensitive measurement of virus replication and spread is used (NA activity). These results underscore that the primary function of the cell substrate as an indicator for virus replication and spread, and furthermore that an acceptable cell substrate should be broadly permissive for replication of both seasonal and non-seasonal influenza viruses to be useful. In this regard, MDCK cells have been confirmed to be a suitable cell substrate for the microneutralization assay.

## Materials and Methods

### Cell Lines

All cell lines were originally obtained from the American Type Culture Collection (ATCC) or European Collection of Cell Cultures (ECACC). The cell lines used were: the human cell lines Caco-2 (human colorectal epithelial cells, HTB-37, ATCC), A549 (human lung epithelial cells, CCL-185 from ATCC), HEp-2 (human epithelia cells, CCL-23, ATCC), and NCI-H292 (human lung epithelial cells, CCL-1848, ATCC); the monkey cell lines Vero (African green monkey kidney epithelial cells CCL-81, ATCC) and LLC-MK2 (Rhesus monkey kidney epithelial cells, CCL-7, ATCC); the mink lung cell line Mv 1 Lu (mink lung epithelial cells, CCL-64, ATCC); and the canine cell line MDCK (canine kidney epithelial cells, 8412903, ECACC). All cell lines were maintained and propagated at the MedImmune Cell Culture facility in MountainView, CA. Cell banks were sterility and mycoplasma tested, and found to be free of detectable microbial agents. Cells were typically seeded in 96-well plates in cell growth medium (Eagle’s Minimum Essential Medium supplemented with 2 mM L-glutamine, 50 µg/ml gentamicin sulfate, and 10% fetal bovine serum). The seeding cell density was 8–80,000 cells per well, depending upon the cell line, to ensure ∼100% cell monolayer confluency after 2 or 3 day incubation at 36°C or 37°C in 5% CO_2_.

### Influenza Viruses

The seven wild type influenza viruses used in the study were obtained from MedImmune Material Management. The viruses used were: two H1N1 strains, A/South Dakota/6/07 (A/SD) and A/California/07/09 (A/CA); two H3N2 strains, A/Uruguay/716/07 (A/UR) and A/Perth/16/09 (A/PTH); the pandemic H9N2 strain, A/Chicken/Hong Kong/G9/1997 (A/HK); and two B strains, B/Florida/04/06 (B/FL, Yamagata lineage) and B/Malaysia/2506/04 (B/MAL, Victoria lineage). Prior to the study all influenza viruses were titered for infectivity on MDCK cells using a fluorescent focus assay (FFA) and stored as single use aliquots at −80°C.

### Monoclonal Antibodies and Polyclonal Sera

Monoclonal antibodies used for immunostaining of influenza infected cells were anti-human influenza A (H1N1, H2N2) from Takara Bio Inc (Cat# M145) for staining H1N1 strains, anti-human influenza A (H3N2) from Takara Bio Inc (Cat# M146) for staining H3N2 strains, anti-human influenza B from Millipore (Cat# MAB8671) for staining B strains and anti-human influenza A nucleoprotein from AbD Serotec (Cat# MCA400) for staining the H9N2 strain. Sheep polyclonal sera against the HA protein used in the MN assay were raised using purified bromelain-cleaved HA from matching strains at MedImmune and are not commercially available. Sheep polyclonal sera against the NA protein used in the microneutralization assay were purchased from National Institute for Biological Standards and Control (NIBSC). The NIBSC sheep anti-NA polyclonal sera, anti-N1 Neuraminidase (Cat# 04/230), anti-N2 Neuraminidase (Cat# 04/258), anti-B/Florida Neuraminidase (Cat# 09/316), and anti-B/Malaysia Neuraminidase (Cat# 05/252) were raised against the H7N1 reassortant virus between A/Equine/Prague/56 (H7N7) and A/New Caledonia/20/99 (H1N1), the H7N2 reassortant virus between A/Equine/Prague/56 (H7N7) and A/Wyoming/3/2003 (H3N2), purified NA from B/Florida/4/2006, and purified NA from B/Malaysia/2506/2004, respectively. Naive sheep polyclonal serum was prepared at MedImmune and pooled from multiple pre-bleeds. All ferret sera used in the study were collected as either pre-bleed or post immunization sera (varies from day 21 to day 42) and used in pairs. Ferrets were immunized intranasally with the matching vaccine strains (*ca*) made at MedImmune and pre- and post-immunization serum collected at the MedImmune Animal facilities.

### Cell Line Screening

The growth properties for all eight cell lines were assessed prior to the study and plating conditions were established to ensure that each cell line would reach ∼100% confluency at the time of virus infection. Each influenza virus used in the study was titered by FFA potency assay. Viruses were serially diluted 10-fold in serum-free virus growth medium (supplemented with 0.1 or 0.5 µg/ml trypsin or none) and the same dilutions were applied in duplicate wells to all cell lines. Based on the MDCK titers, each plate received ∼10,000 FFU sequential ten-fold reduction to limiting dilution of ∼0.01 FFU from row A-G, with Row H received no virus. Infected plates were incubated at 33°C for 1, 3, or 6 days. On Day 1 (18–22 h) post-infection, one plate was processed for infectivity by FFA assay. On Day 3 or Day 6, one plate was checked for CPE under a light microscope. Next, 50 µl of the spent medium was transferred to another reaction plate for NA assay. Finally, the remaining cells were washed and immunostained using type/subtype specific antibodies to assess the HA expression. To minimize day-to-day variability resulting from cells and analysts, and to demonstrate reproducibility and consistency, all setups were repeated 3 times.

### Fluorescent Focus Assay (FFA)

The infectivity of influenza virus preparations were determined using the FFA assay following a MedImmune standard operating procedure. Briefly, confluent cells (MDCK or others) in 96-well plates were infected with the selected influenza virus using 10-fold serial dilutions, starting at approximately 10,000 FFU per well to limiting dilution, and allowed to incubate at 33°C for 18–22 h. At the end of the incubation cells were washed once with phosphate buffered saline (PBS) and fixed with 4% paraformaldehyde in PBS for 15 min at room temperature. The fixed cells were washed and immunostained with anti-HA-specific monoclonal antibodies and infected cells were detected with goat anti-mouse IgG-Alexa 488 conjugates (Invitrogen, Cat# A11017). Fluorescent infected cell foci were enumerated under a fluorescence microscope either manually or using an automated image acquisition instrument (Isocyte®), then converted to titers in fluorescent focus forming units per volume (FFU/ml) or per well.

### Cyotpathic Effects (CPE) and HA Antigen Expression

The replication and spread of influenza virus preparations were determined using cytopathic effects (CPE) and hemagglutinin (HA) expression at 3 and 6 day post-infection. CPE was observed microscopically and HA was monitored by immunofluorescence-staining as described in the FFA assay on days 3 and 6 post-infection. Production of HA antigen and CPE were scored subjectively based on a scale+(<20% or low levels of antigen/CPE),++(20% to 50%, moderate levels of antigen/CPE), or+++(>50%, high levels of antigen/CPE). Wells that showed no spreading (NS) of observed foci (Day 3 post-infection) or foci not detected (ND) were also recorded. For calculation purposes, the subjective scores were assigned numeric values as+(1),++(2),+++(3), NS (0.5), and ND (0) for calulations.

### Neuraminidase (NA) Assay

The virus-associated NA activity in the culture medium was measured by conversion of the fluorogenic reagent 2′-(4-methylumbelliferyl)-α-D-N-acetylneuraminic acid (MU-NANA) using a MedImmune standard operating procedure. Briefly, at selected days post-infection, 50 µl of the spent cell culture medium from each well was transferred to a black 96 well reaction plate and mixed with 50 µl of MU-NANA reaction buffer (250 mM sodium acetate, 88 mM calcium chloride, pH5.5) containing 50 µM MU-NANA substrate. After incubation for 2 h at 37°C, the reaction was stopped with the addition of 50 µl 0.5 M glycine (pH 10.4). The resulting fluorescent intensity was measured using the SpectraMax M5 (Molecular Devices) with settings of 355 nm for excitation and 460 nm for emission.

### Microneutralization (MN) Assay

The MN assay using MDCK cells (or other cell substrates) was performed with minor modification of a MedImmune standard operating procedure. Briefly, ∼100 FFU (as determined in MDCK cells) of selected type/subtype virus was added to 2-fold serum dilutions of antiserum in serum-free virus growth medium. The virus and serum samples were allowed to incubate at room temperature for 30 min. Following virus neutralization, the test samples were transferred onto confluent cell substrates in 96-well plates (200 µl final volume, duplicate wells), and incubated for 1, 3 or 6 days at 33°C. Culture medium was supplemented with TPCK-trypsin at 0.1 µg/ml as required to allow cleavage of HA and spread of virus to adjacent cells. The amount of trypsin used in the MN assay balanced the need to allow virus growth with the propensity of the cell line to detach from the plastic substrate over six days of incubation. At Day 1 post-infection, plates were immunostained as used for the FFA assays and the MN titer for a serum sample was derived based on 50% reduction of the foci counts. At Day 3 and 6 post-infection, the MN titer was determined by NA activity (50% reduction in NA activity) or by HA antigen expression through immunofluorescent staining using type/subtype specific sheep polyclonal sera. The neutralization assay was repeated 3 times (for Day 1 and Day 3 post-infection measurements) or 6 times (for Day 6 post-infection measurements) to ensure an accurate estimate of the neutralization titers. Data collected included the baseline pre-immune neutralization titer and the post-immune titer by strain and antisera for the ferret sera. For the hyper-immune anti-HA and anti-NA antisera, normal (naïve) sheep serum was used as a control. The minimum dilution tested for the normal sheep serum was 6.3 log_2_. The fold rise in titer from baseline to post-immune was calculated by strain and antisera.

## References

[1] European Medicines Agency (2010) Report from scientific workshop on serology assays and correlates of protection for influenza vaccines 29–30 June. 2010: EMA/732806/2010.

[2] Granström M, Voordouw AC (2011) Registration of influenza vaccines for children in Europe. Vaccine 29(43): 7572–5. 21820474 10.1016/j.vaccine.2011.08.017

[3] World Health Organization (2011) WHO Global Influenza Surveillance Network: Manual for the laboratory diagnosis and virological surveillance of influenza. ISBN 978-92-4-154809-0.

[4] Frank AL, Puck J, Hughes BJ, Cate TR (1980) Microneutralization test for influenza A and B and parainfluenza 1 and 2 viruses that uses continuous cell lines and fresh serum enhancement. J Clin Microbiol 12: 426–432. 6260835 10.1128/jcm.12.3.426-432.1980 PMC273601

[5] Li IW, Chan KH, To KW, Wong SS, Ho PL, et al (2009) Differential susceptibility of different cell lines to swine-origin influenza A H1N1, seasonal human influenza A H1N1, and avian influenza A H5N1 viruses. J Clin Virol 46: 325–330. 19801200 10.1016/j.jcv.2009.09.013

[6] Schepetiuk SK, Kok T (1993) The use of MDCK, MEK and LLC-MK2 cell lines with enzyme immunoassay for the isolation of influenza and parainfluenza viruses from clinical specimens. J Virol Methods 42: 241–250. 8390473 10.1016/0166-0934(93)90036-q

[7] Zhirnov O, Klenk HD (2003) Human influenza A viruses are proteolytically activated and do not induce apoptosis in CACO-2 cells. Virology 313: 198–212. 12951033 10.1016/s0042-6822(03)00264-2

[8] Zhirnov OP, Vorobjeva IV, Saphonova OA, Poyarkov SV, Ovcharenko AV, et al (2009) Structural and evolutionary characteristics of HA, NA, NS and M genes of clinical influenza A/H3N2 viruses passaged in human and canine cells. J Clin Virol 45: 322–333. 19546028 10.1016/j.jcv.2009.05.030

[9] Andrewes CH, Elford WJ (1933) Observation on anti-phage sera. I. "The percentage Law.". Brit J Exptl Pathol 14: 367–376.

[10] Chiapponi C, Zanni I, Garbarino C, Barigazzi G, Foni E (2010) Comparison of the usefulness of the CACO-2 cell line with standard substrates for isolation of swine influenza A viruses. J Virol Methods 163: 162–165. 19781571 10.1016/j.jviromet.2009.09.017

[11] Hussain AI, Cordeiro M, Sevilla E, Liu J (2010) Comparison of egg and high yielding MDCK cell-derived live attenuated influenza virus for commercial production of trivalent influenza vaccine: in vitro cell susceptibility and influenza virus replication kinetics in permissive and semi-permissive cells. Vaccine 28: 3848–3855. 20307595 10.1016/j.vaccine.2010.03.005 PMC7172923

[12] Kim JS, Kim SH, Bae SY, Lim CS, Kim YK, et al (2008) Enhanced detection of respiratory viruses using cryopreserved R-Mix ReadyCells. J Clin Virol 42: 264–267. 18467164 10.1016/j.jcv.2008.03.015

[13] El Ahmer OR, Raza MW, Ogilvie MM, Weir DM, Blackwell CC (1999) Binding of bacteria to HEp-2 cells infected with influenza A virus. FEMS Immunol Med Microbiol 23: 331–341. 10225293 10.1111/j.1574-695X.1999.tb01255.x

[14] Hierholzer JC, Castells E, Banks GG, Bryan JA, McEwen CT (1993) Sensitivity of NCI-H292 human lung mucoepidermoid cells for respiratory and other human viruses. J Clin Microbiol 31: 1504–1510. 8314992 10.1128/jcm.31.6.1504-1510.1993 PMC265568

[15] Lam WY, Tang JW, Yeung AC, Chiu LC, Sung JJ, et al (2008) Avian influenza virus A/HK/483/97(H5N1) NS1 protein induces apoptosis in human airway epithelial cells. J Virol 82: 2741–2751. 18199656 10.1128/JVI.01712-07 PMC2258969

[16] Benne CA, Harmsen M, De Jong JC, Kraaijeveld CA (1994) Neutralization enzyme immunoassay for influenza virus. J Clin Microbiol 32: 987–990. 8027355 10.1128/jcm.32.4.987-990.1994 PMC267167

[17] Orstavik I (1981) Susceptibility of continuous lines of monkey kidney cells to influenza and parainfluenza viruses in the presence of trypsin. Acta Pathol Microbiol Scand B 89: 179–183. 6274142

[18] Genzel Y, Reichl U (2009) Continuous cell lines as a production system for influenza vaccines. Expert Rev Vaccines 8: 1681–1692. 19943763 10.1586/erv.09.128

[19] Govorkova EA, Murti G, Meignier B, de Taisne C, Webster RG (1996) African green monkey kidney (Vero) cells provide an alternative host cell system for influenza A and B viruses. J Virol 70: 5519–5524. 8764064 10.1128/jvi.70.8.5519-5524.1996 PMC190510

[20] Usuba O, Schulman JL, Deatly AM, Bona CA, Moran TM (1990) New method for titration of virus infectivity by immunostaining. Viral Immunol 3: 237–241. 2175196 10.1089/vim.1990.3.237

[21] Barenfanger J, Drake C, Mueller T, Troutt T, O'Brien J, et al (2001) R-Mix cells are faster, at least as sensitive and marginally more costly than conventional cell lines for the detection of respiratory viruses. J Clin Virol 22: 101–110. 11418357 10.1016/s1386-6532(01)00171-8

[22] Hamilton SB, Wyatt DE, Wahlgren BT, O'Dowd MK, Morrissey JM, et al (2011) Higher titers of some H5N1 and recent human H1N1 and H3N2 influenza viruses in Mv1 Lu vs. MDCK cells. Virol J 8: 66. 21314955 10.1186/1743-422X-8-66 PMC3046928

[23] Schultz-Cherry S, Dybdahl-Sissoko N, McGregor M, Hinshaw VS (1998) Mink lung epithelial cells: unique cell line that supports influenza A and B virus replication. J Clin Microbiol 36: 3718–3720. 9817906 10.1128/jcm.36.12.3718-3720.1998 PMC105273

[24] Hassantoufighi A, Zhang H, Sandbulte M, Gao J, Manischewitz J, et al (2010) A practical influenza neutralization assay to simultaneously quantify hemagglutinin and neuraminidase-inhibiting antibody responses. Vaccine 28: 790–797. 19887135 10.1016/j.vaccine.2009.10.066

